# Treating transsexuals in India: History, prerequisites for surgery and legal issues

**DOI:** 10.4103/0970-0358.59287

**Published:** 2009

**Authors:** Richie Gupta, Anil Murarka

**Affiliations:** Sunderlal Jain, Max and Maharaja Agrasen Hospitals, Delhi, India

**Keywords:** Gender affirmation, sex change, transsexuality

## Abstract

Authors in their clinical practice came across transsexual patients, who were determined to get their gender affirmed by undergoing a change of sex. This motivated the authors to review the literature extensively regarding transsexualism and report their experience. Opinions were taken from legal luminaries practicing in related fields. They also took inputs from several patients who were at various stages of psychiatric analysis and hormone therapy and also those, who had completed their treatment procedures. A paucity of the Indian inputs in medical literature concerning transsexualism was noted by the authors They also found deficiencies in the Indian Law, as applied to the individuals undergoing gender affirmation surgery (GAS). In this paper they have enumerated these deficiencies. Though GAS has been legally allowed in U.K. since 1967, in America since 1972, and in various other countries, Indian Laws are silent on the issue. An Indian surgeon dealing with transsexual patients is faced with a number of issues like consent for the procedure, safe guarding the surgeon or gender team from future litigation. Another issue is postoperative sexual and legal status of the patient. Present Indian Laws regarding marriage, adultery, sexual and unnatural offences, adoptions, maintenance, succession, labour and industrial laws will require modifications when dealing with these individuals and protecting their rights. Authors have tried to deal with all these issues that an individual surgeon faces when he manages a transsexual patient.

## TRANSSEXUALISM-THROUGH THE AGES

Indian mythology has in it, many references to altered sexual states. The name Ardhanarishwara[1–3] refers to God, who is half man and half woman, an androgynous deity. In various versions of Ramayana, there is reference to King Ila,[1] who spent half his life as man and half as woman. In Mahabharata, Arjuna,[1] one of the fiercest warriors of his time, spent a year of his life in intersexed condition. There is also reference to King Bangasvana,[1] who was changed into a woman by Lord Indra, whom he had offended. Another reference during Mahabharata is to Shikhandini.[4] He was born female, but raised like a man and trained in warfare. After an encounter with a Yaksha, Shikhandini came back as a man, was called Shikhandi and fathered children.

The transsexual phenomenon has been mentioned from time to time in recorded history. Pharaoh Hatsepshut,[5–7] a female who ruled Egypt from 1479-1458 BC, invented a hybrid gender so that she could rule. At that time, a Pharaoh was by definition, male. King Henry III of France in 16^th^ century frequently cross dressed and was often referred to as ‘Her Majesty’ by courtiers.[5] In 17th century, Queen Christina of Sweden gave up the throne, cross dressed and renamed herself ‘Count Dohna’.[5,8] In 1673, French explorers, Louis Joliet and Jacques Marquette, discovered a group of Illini Indians, who dressed and lived as women.[5] The Illini termed these men Ikoneta, while the French called them Berdache. Chevalier D'Eon, a famous spy and ambassador in 18th century was born as a male (Charles) but lived a significant part of his/her life as a woman, thus giving rise to the expression ‘eonism’.[5,9] Billy Lee Tipton (born Dorothy Lucille Tipton, 1914-1989) was an American Jazz Pianist and Saxophonist. He became a subject of interest posthumously when it was revealed that this thrice married musician, who had two adopted sons was in fact, a female.[5,10] Transsexual people differ from transvestites who merely wear the clothing of an opposite sex. They also differ from Hijras as found in the Indian society, as these mostly suffer from childhood castration,[1] there being rare cases of intersex.

In 1932, ‘Man into Woman’, the story of Lili Elbe's life, male to female transition and sex reassignment surgery was published.[5,11] She was born as Ejnar Mogens Wegener in Denmark. She underwent five operations including ovarian and uterine transplants and probably died due to complications from rejection process.[5,12] She underwent most likely, procedures with no scientific backing. In 1945, Sir Harold Delf Gillies widely regarded as the Father of Modern Plastic Surgery,[5,13] together with Ralph Millard carried out the first scientifically performed sex change procedure of a woman Laura Dillon to man. He was renamed Michael Dillon.[5,13,14] Later he did the UK's first male to female operation (Robert to Roberta Cowell).[5,13–16] In 1967, a change in British Laws allowed Charing Cross Hospital to begin performing the sex change surgery. In 1972, American Medical Association sanctioned sex change surgery as the treatment for transsexualism.[5] Japan allowed first legal sex change operation from female to male in 1998.[5]

Harry Benjamin (1885-1986)[5,17] recognized transsexualism, treated hundreds of patients and established the modern scientific management of this condition. He wrote many books, especially ‘The Transsexual phenomenon’ in 1966. Many of his patients went on to become celebrities. These included April Ashley,[5,18,19] Christine Joregenson,[5,17,20] Coccinelle[17,21] and Roberta Cowell.[5,13–16] Norman Fisk[5,22] in 1973 coined the term ‘Gender Dysphoria Syndrome’. In 1979, Harry Benjamin's International Gender Dysphoria Association (HBIGDA) was founded. They established the transsexual Standards of Care (SOC's) and established the criteria for diagnosis, management and surgery. This was periodically revised and the most recent version (sixth) was published in February 2001. HBIGDA itself is presently known as ‘World Professional Association for Transgender Health’ (WPATH). In 1980, American Psychiatry Association listed transsexualism as an official disorder in DSM-III. The diagnosis was changed to ‘gender identity disorder’ in DSM -IV. The disorder is now likely to be removed from DSM and considered a variation of normal. In 1998, Brain material provided by the Netherlands Brain Bank demonstrated transsexualism to be a pre -birth medical condition and not a state of mind.[23]

In 1968, International Olympic Committee first started to test the chromosomes of athletes to prevent transsexuals from competing.[5] In the famous Corbett versus Corbett judgement (1970), April Ashley's marriage was declared null and void despite having undergone sex change surgery.[5,18,19] In 1976, Tennis Ace Renee Richards was barred from entering women's tournament.[5,24] She won the following legal battle. Christine Goodwin versus the U.K. case (1999 -2002) paved the way for Gender Recognition Act to become U.K. Law.[5,25] The transsexuals were now legally allowed to marry. In 2003 IOC allowed transsexuals to compete in Athens Olympics as the members of their new sex provided, they were legally recognized, had undergone sex change operation, and had received at least 2 years of hormone therapy.[5,26] Felicity Huffman won a Golden Globe Award (2006) and was nominated for Oscar for her role in the movie ‘Transamerica’.[5,27] She played the role of male to female transsexual Sabrina Bree Osbourne, who while in the midst of her transition, discovered that she had fathered a son earlier from a one night stand. Her psychiatrist asked her to deal with this situation before permitting her to complete her procedure. It portrays many of the problems faced in everyday life by transsexuals. Some female to male transsexuals who interrupt hormone treatment and have functioning ovaries can become pregnant. Thomas Beatie chose to become pregnant as his wife was infertile.[5,28] He was registered as a male in state of Oregon. Thus, he became the first legal male pregnancy on record, although Matt Rice bore a child by artificial insemination way back in 1999.[5,29,30]

## SEX, GENDER AND TRANSSEXUALISM

To understand transsexuality, we have to understand the difference between sex and gender. While ‘sex’ represents physical differentiation as male or female, indicated by the external appearance of genitalia and the presence of gonads, ‘gender’ is the psychological recognition of self, and wish to be regarded by others, as fitting into the social categories such as boy/man or girl/woman. In short, sex is what one is seen as (external appearance as male/ female) and gender being the identity is what one feels (playing the role of and living the life of male/ female).[31] The feeling of an incongruence between sex and gender is termed gender dysphoria. Transsexualism is the most extreme form of this disorder. These individuals feel themselves to be trapped in the wrong body (transsexual phenomenon). They need to adapt their phenotype with hormones and surgery to make it congruent with their gender identity.[32] There are many terms coined for sex change surgery, such as sex reassignment surgery, gender reassignment surgery, sex reconstruction surgery, sex affirmation surgery, feminizing or masculanizing genitoplasty. There is a broad feeling that gender, being genetically hardwired into brain, is not subject to reassignment. It can at best be affirmed by bringing the external appearance (sex) of the person to become congruent with his/her gender.[33] Thus, the most appropriate term may be gender affirmation surgery (GAS). Transsexuals undergoing female to male (FTM) transition are known as transmen and those undergoing male to female (MTF) transition are known as transwomen.

## PREVALENCE, NEURAL & GENETIC BASIS OF TRANSSEXUALISM

The prevalence of transsexualism in the Netherlands is estimated to be 1:11900 males and 1:30400 females.[34] In Sweden, it is 1:37000 among males and 1:30000 in females. The estimates for USA are 1:100000 for males and 1:400000 for females. No definite figures are available for India. From an early age, people develop their gender identity, a sense of being male or female. Transsexuals, however identify themselves with a psychological sex, opposite to their physical sex and hence feel trapped in the wrong body. Recent research indicates that there may be a neural and genetic basis for this phenomenon. Transsexualism is strongly associated with unusual neurodevelopment of brain at the foetal stage. Many sexually dimorphic nuclei have been found in the hypothalamus.[23,35–37] One of these is the central subdivision of Bed nucleus of Stria Terminalis (BSTc). In human males, the volume of this nucleus and its number of neurons is twice compared to females.[23,38,39] In transsexuals, this nucleus had a sex reversed structure, i.e. in transwomen, it was similar to female controls and vise versa.[23] This would suggest a neurologic basis to transsexualism. Eric Vilain[40] discovered 54 genes that link to gender. 18 of these genes are produced at a higher level in males and 36 in females. There is activation of these genes, earlier to the SRY genes in Y chromosome, responsible for testosterone production and hence masculinization of foetus. This refuted the earlier held belief that fetal brain only needed exposure to testosterone to become male. In another study DNA samples were collected from 112 transwomen and genetic differences were compared with non transsexuals.[41] It was discovered that transwomen were more likely to have longer version of a gene which is known to modify the action of testosterone. These genetic differences may reduce testosterone action and reduce the masculinization of brain during foetal development. Hence there is a genetic basis for gender and this identity evolves even before the production of sex hormones and sexual differentiation as a male or female.

## PREREQUISITES FOR GENDER AFFIRMATION SURGERY[31,34]

Before undertaking a GAS, certain conditions must be met and criteria satisfied. These are:

### A firm diagnosis for the transsexual condition. The criteria for these are:

**A)**

1. A sense of discomfort and inappropriateness about one's sex.
2. A wish to be rid of one's genitalia and the desire to live life as a member of the opposite sex.
3. This discomfort/disturbance has been continuously present for a minimum of 2 years and is not limited to a period of stress.
4. An absence of physical intersex or genetic abnormality.
5. Absence of a mental disorder such as schizophrenia.

### Differential diagnosis for the condition may be-

**B)**

1. Classic trans -sexual.
2. Gender dysphoria syndrome, (formerly effeminate homosexuality)
3. Gender dysphoria syndrome, (formerly transvestitism)
4. Gender dysphoria syndrome, psychosis.
5. Gender dysphoria syndrome, psycho -neurotic sociopathy.
6. Gender dysphoria syndrome, inadequate and schizoid personality.

If the final diagnosis of patient is ‘Classic Transsexual’, he/she may be a candidate for GAS. The management of transsexuals is guided by Harry Benjamin International Gender Dysphoria Association's (HBIGDA) standards of care (SOC's) sixth version published in February 2001. HBIGDA itself is now known as ‘World Professional Association for Transgender Health’ (WPATH).

### Requirement of referral letters from mental health professionals

**C)**

1. **Letter from one mental health professional is required for instituting hormone therapy, or for breast surgery.**
  One letter from a mental health professional, including the seven points mentioned in point D, written to the physician who will be responsible for the patient's medical treatment, is sufficient for instituting hormone therapy or for a referral for breast surgery (e.g., mastectomy, chest reconstruction, or augmentation mammoplasty).
2. **Letters from two mental health professionals are required before carrying out Genital Surgery.**
  - Genital surgery for male to female transsexuals includes - orchiectomy, penectomy, clitoroplasty, labiaplasty or creation of a neovagina.
  - Genital surgery for female to male transsexuals includes - hysterectomy, salpingo -oophorectomy, vaginectomy, metoidioplasty, scrotoplasty, urethroplasty, placement of testicular prostheses or creation of a neophallus.

### The documentation letter from mental health professional should mention these seven points -

**D)**

1. A description of patient's general identifying characteristics.
2. Initial and evolving gender, sexual and other psychiatric diagnoses.
3. Duration of professional relationship with the patient, number of consults, the type of psychotherapy or evaluation that the patient underwent.
4. Specifying the eligibility criteria that have been met and the rationale for prescribing hormone therapy or surgery.
5. Degree to which the patient has followed the Standards of Care till the time of writing, and the likelihood of future compliance.
6. Whether the mental health professional is part of a gender team.
7. That the mental health professional would like to receive a phone call from the gender team confirming the authenticity of this letter.

**An appropriate period of hormone therapy,** usually 6 months. In many transwomen (M to F), a formal breast augmentation is not required as sufficient hypertrophy occurs on hormone therapy.

**E)**

### a) Why is hormone therapy required -

Trans-sex hormonal treatments are important for a smooth gender transition, both physically and psychologically in selected individuals with gender identity disorders. In the absence of indigenous source, hormone therapy is medically necessary for a satisfying role in the patient's new sex. When physicians administer androgens to biologic females and estrogens, progesterone, and testosterone -blocking agents to biologic males, patients feel and appear more like members of their preferred sex.

### b) Eligibility criteria for hormone therapy -

Administration of hormones is not to be taken lightly because of associated medical and social risks. The criteria are -

1. Age 18 years or above.

2. In depth knowledge of what hormones can achieve medically, their social benefits and risks.

3a. A documented real - life experience of at least 3 months prior to the administration of hormones or

3b. A period of psychotherapy of a duration specified by the mental health professional after the initial evaluation (usually a minimum of 3 months).

**Consent Forms** - It is advisable to convert the b, c and d (written below) into affidavits by paying the Notary or Court Fee and getting the counter signatures of a Notary or Magistrate, respectively.[42] This means that now the state is a witness.

**F)**

**Figure 1:**
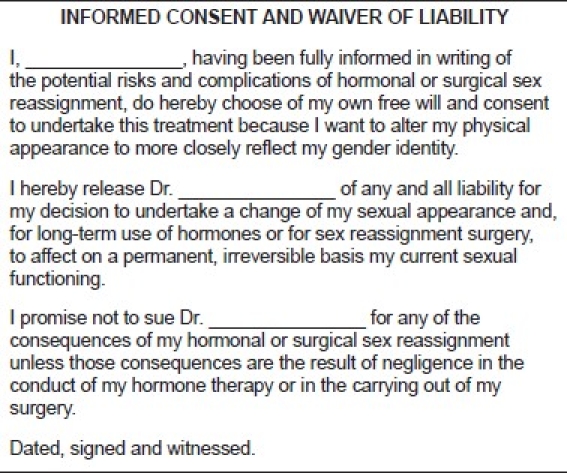
Waiver of liability form
- Spousal Release Form [Figure 2]: This is used if the patient is married.

- Procedure - specific consent form: It includes a description of the surgical procedure. It also mentions the surgical and anaesthetic complications that may occur. This also includes an estimate of the procedure.
- Waiver of Liability Form [Figure 1]: It contains a waiver of liability from the change in sexual appearance, long term use of hormones and permanent, irreversible change in current sexual functioning.

**Figure 2:**
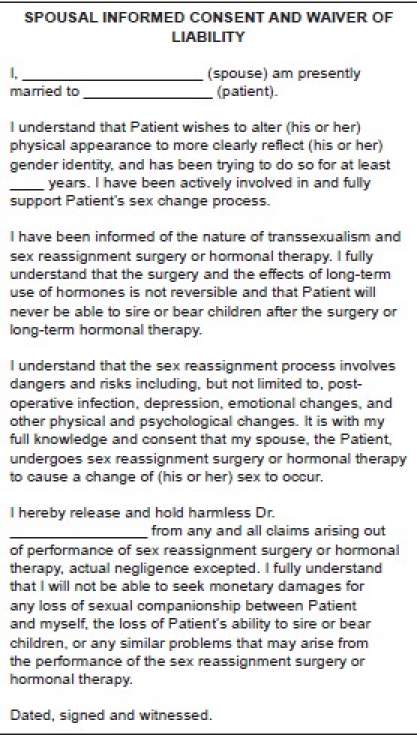
Spousal release form
- Parental Consent Form [Figure 3]: This is used if patient is less than 20 years of age. Currently the management of transsexuals is not started before the age of 18 years except in the Netherlands[33,43] (due to the pioneering work of Dr. Cohen Kettenis) and otherwise under exceptional circumstances.[44]

**Figure 3:**
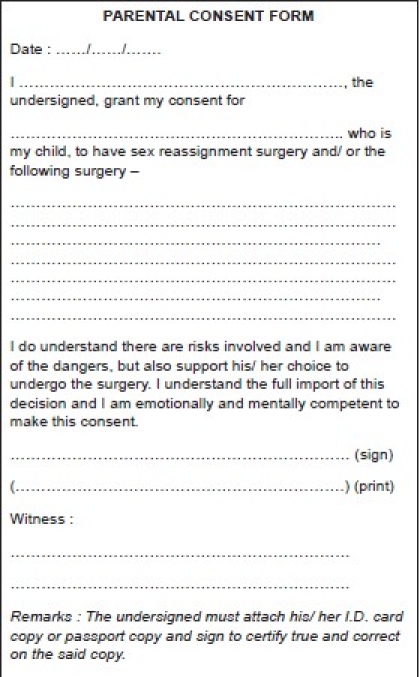
Parental consent form. Used if the patient is above 18 years and below 20 years of age.

## POSTOPERATIVE ISSUES CONCERNING THE PATIENT

- Change of Name
- Change of Name and Sex in various certificates
- Change of Name and Sex in Identity Cards and Passport.

## LEGAL ISSUES REGARDING GENDER AFFIRMATION SURGERY IN INDIA[45]

In India, though there is an increasing incidence of GAS, there is no legal precedent.

GAS involves changes in identity and sex of the person. Consequently, the existing laws in their present form are inadequate when dealing with the rights of transsexuals. Some years back, the sex change operation of Aparna to Ajay Mafatlal, brought some of these issues into prominence. The following laws may require modifications -

### a)  The Penal Code 1860:

1. The definition of ‘rape’ may require a change.
2. Section 377 IPC, dealing with unnatural offence.
3. The definitions of ‘wife’, ‘husband’, ‘adultery’, as incorporated in Sections 497, 498 and 498A IPC.
4. The definition of ‘wife’ as contemplated in Section 125 CrPC and Laws regarding maintenance.

### b)  Personal Laws:

1. Hindu Marriage Act and all personal Laws relating to marriage. The present Laws will not be adequate in questions of maintenance, grounds for divorce and custody of children.
2. Hindu Adoptions and Maintenance Act 1956.
3. Hindu Succession Act, especially Sections 8, 14 and 23.
4. Labour and Industrial Laws, especially Workmen's Compensation Act 1923, Factories Act 1948 and reservation of jobs on the basis of sex.

### c)  Taxing Statutes:

Various beneficial provisions like tax exemptions available only to women, under the Income Tax Act.

## CONCLUSIONS

An Indian surgeon dealing with Transsexual patients is faced with a number of problems. Though GAS has been legally allowed in U.K. since 1967, in U.S.A. since 1972, and in various other countries, Indian Laws are silent on the issue.

Another aspect is the consent for the procedure and safe guarding the surgeon or the gender team from future litigation. Though waiver of liability, spousal release and parental consent forms are available, there is an absence of legal backing. In view of this situation, it is better to convert these consent forms into affidavits by paying the appropriate court or notary fee and getting these signed by a magistrate or notary. This means that ‘the state’ is now a witness to this agreement.[42] At completion of GAS, a Gender Certificate should be issued to the patient by the gender team consisting of the operating surgeon, psychiatrist and endocrinologist.

There is also the issue of postoperative sexual and legal status of the patient. Present Indian Laws regarding marriage, adultery, sexual offences, unnatural offences, adoptions, maintenance, succession, labour and industrial laws will require modifications when dealing with these individuals and protecting their rights, as detailed above.

Postoperatively, the patient faces issues of change in name, birth certificates, school and college certificates and identity cards. Tista Das,[46] a transwoman is the only transsexual to have a valid Voter Identity and Ration Card in India. Abroad, there is a move towards unisex identity cards by eliminating reference to person's sex in these documents. In India, however, the battle is long and lonely. This situation can be amended by new legislation. There can be a nodal authority for dealing with transsexual patient. On presentation of Gender Certificate by the individual, this authority can then retain previous certificates and issue new certificates to the individual.

Transsexualism has been present since ages in the world and also, in India. Authors have tried to identify medical, legal and legislative challenges present before the Plastic Surgeon and his gender team, in dealing with the issues concerning transsexuals. They suggest their solutions for these issues based upon their experience in managing these patients, as well as the extensive review of the Medical and Legal Literature.
